# Examining associations between anxiety and cortisol in high functioning male children with autism

**DOI:** 10.1186/1866-1955-5-32

**Published:** 2013-11-11

**Authors:** David M Simon, Blythe A Corbett

**Affiliations:** 1 Department of Psychiatry, Vanderbilt University, 230 Appleton Way, 37203 Nashville, TN, USA; 2 Vanderbilt Kennedy Center, Nashville, TN, USA

**Keywords:** Autism, Anxiety, Stress, Cortisol, Self-report

## Abstract

**Background:**

Autism spectrum disorder (ASD) is characterized by deficits in communication and social ability, as well as restricted interests and repetitive behavior. Anxiety is a persistent anticipation or apprehension about one or more situations to which a person is exposed, and affects many people, including children with ASD. Stress, by contrast, is a response to situations that are threatening, uncontrollable, or unexpected. Indices of anxiety are often measured through informants, with parents and teachers serving as the primary sources of reported anxiety in children. However, self-report measures exist, allowing current (state) and persistent (trait) anxiety to be assessed. The current study was designed to evaluate whether children with autism could identify their own levels of anxiety and the degree to which these levels were associated with symptom profile and physiological arousal.

**Methods:**

Self-reported state and trait anxiety were collected during exposure to different stress paradigms for 40 children (21 typically developing, 19 with autistic disorder) and compared to parent reported social ability (Social Responsiveness Scale) and stress responsivity (cortisol).

**Results:**

Significant differences were found between typically developing and children with autism for both state and trait anxiety across all conditions. Associations were identified between severity of parent-reported social impairment and both types of self-report anxiety. No relationship was found between stress (salivary cortisol) and anxiety in children with autism.

**Conclusions:**

Children with autism are able to consistently report their persistent level of anxiety symptoms in stressful situations of benign character. Therefore, the inclusion of such measures may be useful in identifying and tracking symptoms in children with autism under appropriate circumstances.

## Background

In addition to the primary impairments in reciprocal social communication and repetitive, restricted behaviors in autism spectrum disorder [1], there are frequent reports of comorbid anxiety disorders such as phobias, social anxiety disorder, and obsessive-compulsive disorder [2]. Anxiety, defined here as a persistent anticipation or apprehension regarding one or more situations to which a person is exposed, is an acceptable and typical reaction to some events. Clinical anxiety disorders, however, are characterized by excessive worry and distress [3], and are among the most commonly reported childhood disorders with rates ranging from 3% to 24% in the general population [4]. High frequencies of anxiety are found in autism as well, with reported rates of comorbid anxiety disorders as high as 42% to 55% [5,6], and as many as 84% showing some level of impairing anxiety [7-10]. The nature of the link between anxiety symptoms and autism symptomology is unclear, with some investigations showing a lack of correspondence [11,12], while others show relations to specific symptoms such as hypersensitivity to sensory stimuli [13]. Links with comorbid conditions such as disruptive behavior have also been demonstrated [14]. Moreover, beyond the diagnostic impairments of autism, parents frequently report anxiety related issues to be a major source of challenge they face [15].

Longitudinal and developmental neuroscience studies reveal that alongside cognitive and neural development, anxiety often follows a developmental trajectory starting early in life and proceeding into adolescence and adulthood [16,17]. For example, in a study exploring error related negativity, an event related potential component shown to be more prominent in adult anxiety, Meyer and colleagues [18] reported changes in anxiety as a function of age in children 8 to 13 years of age. In children with autism, there is evidence that age is a significant factor not only in the prevalence of anxiety but also the type of anxiety present [2]. It is important to acknowledge, however, that overall diagnostic stability of anxiety over extended periods of time is, at best, moderate in children and adolescents [19,20]. While these emerging studies support consideration of cognitive development when investigating anxiety there is a paucity of knowledge pertaining to intellectual functioning and its relationship to anxiety in children with neurodevelopmental disorders such as autism. Based on recent findings there appears to be a relationship between level of cognitive function and anxiety; specifically, intellectually high functioning children with autism are at particular risk for anxiety disorders [21]. The need for accurate tools for rapid and accurate assessment of anxiety in this population is in many respects drawn from the necessity of isolating these developmental, maturational, and cognitive factors in respect to their contributions to pathological anxiety.

Cognitive ability and impairment in autism are distinct; thus, severity of impairment in other core domains, such as social difficulty, can be major contributors to anxiety. Additionally, while autism symptom expression and social anxiety symptoms have been associated in early life, including non-autistic children with clinical anxiety disorders displaying autism-like symptoms and developmental traits [22], the relationship as children age becomes more complex and less understood. Children with autism and higher anxiety display more overall repetitive behaviors, while showing a specific positive relationship between anxiety levels and desire for environmental consistency. In contrast, children with autism and lower anxiety display a positive association between anxiety and sensory motor repetitive behaviors [23]. There is also evidence that severity of excessive anxiety is associated with decreased quality of life in children with autism, as well as those with typical development [24].

Although a certain amount of anticipatory anxiety is considered adaptive, it can become pathological when the worry is excessive or uncontrollable and begins to exert strong influences on behavior and cognition [3]. Increased arousal to perceived novel, uncontrollable, and unexpected or socially threatening situations can also be adaptive [25,26] unless the response is frequent, intense, or in response to a benign situation. In other words, both the anticipation of potentially threatening events (anxiety) as well as the response to such events (stress) can be appropriate. Disorders marked by atypical perception or response to environmental stimuli, such as ASD, may therefore contribute to inappropriate and pathological activation of arousal systems.

One of the primary stress systems, the hypothalamic-pituitary-adrenocortical axis (HPA) responds consistently to perceived novel or unfamiliar situations. Cortisol is a glucocorticoid associated with activation of the HPA axis and can serve as an important biomarker of stress to a variety of different stimuli [27,28]. Previous research has suggested that many children with autism exhibit dysregulation of the HPA system evidenced by enhanced responsivity in different social and non-social situations [29-34]. Higher cortisol levels have been reported in children with autism in response to non-social stimuli including exposure to medical procedures such as phlebotomy [34]. Social scenarios have also resulted in activation of the HPA axis and subsequently elevated cortisol during school integration [33], social interaction with peers on a playground [30,35], and engagement with unfamiliar children [32]. However, not all social stressors are salient for youth with ASD, as a widely recognized social evaluative stressor, the Trier Social Stress Test - Child Version (TSST-C) [36], fails to provoke a stress response in participants with autism [30,31,37,38]. In typically developing children, longitudinal cortisol measurements have shown that persistence of anxiety disorders is associated with increased diurnal cortisol levels, as well as a reduced cortisol awakening response [39], indicating that excessive long-term (trait) anxiety can contribute to increased physiological stress. Despite this relationship, anxiety and stress can sometimes be disassociated, particularly in conditions involving acute (state) anxiety. It has been shown that levels of anxiety and stress are frequently distinct in children with and without autism [31,32]. This lack of a direct biological corollary means that measuring anxiety is often accomplished via proxy measures such as parent report, to the exclusion of more direct self-report measures, as the reliability of the latter has been questioned. Specifically, it has been argued that individuals with autism may not be able to reliably or accurately assess their personal anxiety or affective states [40-42]. This has, in part, been attributed to deficiencies in self-referential cognition [43] as well as deficits in identifying cognitive and emotional states frequently referred to as 'theory of mind’ [44,45]. Some examinations of informant agreement between parents and their children with autism have shown meaningful agreement [46,47] comparable to typically developing youth [48,49]. However, others have shown poor overall agreement supporting the assertion of deficits in self-report ability in this population [40-42].

It should be noted that, while self-reports have been questioned, parent reports are also inconclusive. Recent studies in adolescents with autism suggest discrepancies across informants (parents versus self-reports) for social functioning [50] and quality of life [51]. Importantly, in regards to anxiety symptoms in youth with autism there appears to be consistency across multiple informants (parent, teacher and self-report) regarding the assessment of psychiatric symptoms [52] as well as in response to treatment [53]. These reports are comparable to studies of typically developing children that also show multi-informant consistency [48]. While there have been few studies considering the ability of children with autism to self-report mood states, investigating the reliability and stability of self-reported anxiety in the population is a necessary step for future use of self-report measures.

As part of comprehensive studies examining biobehavioral profiles of children with autism parent report, self-report, and physiological stress data were collected during play with peers [35], exposure to a mock magnetic resonance imaging (MRI) scanner [54], and social evaluation [31]. This repeated participation offered a unique opportunity to investigate relationships between anxiety and stress. Specifically, we examined: (1) the long-term stability of self-reported state and trait anxiety in autism over three stress paradigms; (2) the relationship of self-reported anxiety to parent-reported functioning; and (3) the association between reported anxiety and physiological stress responses. Finally, the exposure to different paradigms afforded the examination of differences based on the social value of stressors, with the MRI serving as a non-social stressor [55], the playground as a benign social stressor [35], and the TSST-C [56] serving as a measure of social evaluative threat. We sought to determine whether the social or non-social nature of the administered stressor influenced the relationship between self-reported anxiety and the physiological stress response as measured by salivary cortisol. We hypothesized that: (1) the children with autism would show consistency in self-reported state and trait anxiety across the paradigms; (2) self-report anxiety would be negatively correlated with parent report of social functioning such that higher levels of anxiety would be associated with lower social functioning; and (3) self-reported anxiety would be correlated with physiological stress indicated by salivary cortisol levels.

## Methods

Participants were recruited through distributed flyers and direct phone contact from a subject tracking system for a playground study examining HPA reactivity to social stressors in children with autism. After completion of the initial playground paradigm participants were recruited by phone for up to two additional studies investigating reactivity to both social and non-social stressors. Only a portion of the participants chose to return for each of the additional studies. The two additional studies had an average latency of 6 months from the initial playground study, with a range of 4 to 9 months. Participants provided saliva samples at 20-minute intervals for later analysis examining salivary cortisol levels at each of the three different stress paradigms described below. Additionally, two separate self-report measures of anxiety were completed at each study session by the participants, while a parent-report measure of social ability was collected at the initial playground session.

### Participants

The playground study consisted of 40 healthy, age matched, unmedicated, prepubertal, male children 8 to 12 years of age. A total of 19 participants were diagnosed as having autistic disorder, and 21 were typically developing. Diagnosis was based on the *Diagnostic and Statistical Manual of Mental Disorders*, 4th edition (DSM-IV) criteria [3] and established by all of the following: (1) a previous diagnosis by a psychologist, psychiatrist, or behavioral pediatrician with autism expertise; (2) clinical judgment and (3) corroborated by a total score on the social-communication scale of the Autism Diagnostic Observation Schedule (ADOS) [57], administered by research-reliable personnel, with a total score at or above the autism threshold for Module 3. Diagnostic and IQ measures were completed together at a separate study visit preceding playground participation. Race/ethnicity of participants was reported by parents as follows: Caucasian 25 (62.5%), Hispanic 6 (15%), African-American 2 (5%), Asian-American 2 (5%), and other 1 (2.5%). Four participants (10%) had no reported race/ethnicity, or had no available data.

A portion of the participants from the playground sample, consisting of 15 (71%) of the typically developing children and 11 (58%) of the children with autism, participated in an additional study using a mock MRI as a non-social stressor. A second subset of the playground sample, including 16 (76%) of the typically developing children and 13 (68%) of the children with autism, participated in the TSST-C, a social stress paradigm. Ten (48%) of the typically developing children and five (26%) of the children with autism from the original playground sample participated in both the mock MRI and TSST-C. Study visits were ordered as follows: playground first, followed by MRI, and finally TSST-C. Additional demographic information is included in Table 1.

**Table 1:** Demographic information

**Sample**	**Variable**	**Autism mean**	**Autism SD**	**Typical mean**	**Typical SD**	***F***	***P *** **value**
Playground	IQ	98.56	17.95	120.80	12.35	20.14	<0.001
Playground	AGE	9.99	1.29	9.92	1.49	0.001	0.99
MRI	IQ	101.92	15.35	122.07	13.101	13.55	0.001
MRI	AGE	10.52	0.94	9.83	1.58	1.773	0.195
TSST-C	IQ	92.92	12.71	123.25	10.97	45.80	<0.001
TSST-C	AGE	9.78	1.12	9.55	1.48	0.280	0.653

*TSST-C* Trier Social Stress Test - Child Version.

Informed written consent was obtained from parents and verbal assent was obtained from research participants prior to inclusion in the study. Participants were told they would be playing on a playground with peers for the playground paradigm or that they would be participating in a school-like performance task in the TSST-C paradigm [56]. Participants in the mock MRI condition were informed that they would be 'practicing’ MRI procedures in a mock scanner. The University Institutional Review Board approved the study, which was conducted in compliance with the Code of the Ethical Principles for Medical Research Involving Human Subjects of the World Medical Association (Declaration of Helsinki).

### Diagnostic and neuropsychological methods

The Autism Diagnostic Observation Schedule (ADOS; [58]) is a semistructured interview designed to assess behaviors indicative of autism. The ADOS was administered by an independent and research reliable psychologist, and has been validated in autistic populations.

The Wechsler Abbreviated Scale of Intelligence (WASI; [59]) is a measure of cognitive ability that was used to obtain a quick and reasonable estimate of a child’s intellectual functioning. An estimated IQ ≥70 was required for participation in the study. The WASI is validated for typically developing individuals.

The Social Responsiveness Scale (SRS; [60]) is a 65-item questionnaire completed by care providers to quantitatively assess the burden of autistic traits in their child. It provides a total score as well as five treatment scales (Social Awareness, Social Cognition, Social Communication, Social Motivation, and Autistic Mannerisms). The SRS has been used in several behavioral and pharmaceutical treatment studies in autism for example [61,62], and has been validated in both typically developing children and children with autism, with Cronbach’s α ranging from 0.71 to 0.89. The total raw scores were used in the analysis.

The State Trait Anxiety Inventory for Children (STAIC; [63]) is a 40-item self-report form used to assess current (state) as well as long-term and persistent (trait) anxiety in children. It has been used extensively as a method of collecting self-report anxiety data in children and yields comparable results to other self-report and parent-report anxiety measures [64]. The measure has been validated in typically developing individuals with a Cronbach’s α of 0.91. Raw scores for each type of anxiety were used in the analysis.

The Multidimensional Anxiety Scale for Children (MASC; [65]) is a 39-item anxiety self-report across clinically significant symptom domains designed for use in children ages 8 to 19. The MASC has been validated in typically developing children, with a Cronbach’s α of 0.90 for the full measure, 0.82 for the social anxiety subscale, and 0.66 for the coping subscale. The complete form was used to gather information across all subscales for all participants.

### Playground paradigm

**Figure 1:**
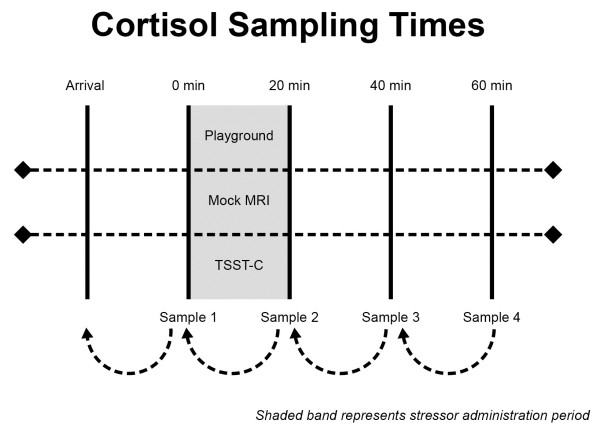
Timeline for saliva sampling across the different stressors.

The playground social stress protocol was the positive social stressor in this study. Participants were asked to play on an enclosed 18.2 × 18.2 m playground with two other age and gender matched children. The 20-minute play period was subdivided into periods of prescribed free and cooperative play was facilitated by a confederate child on the playground communicating with the research staff via concealed audio technology [30]. A total of four salivary samples were obtained, including (PS1) a sample taken 15 minutes after arrival, immediately prior to the playground peer interaction, (PS2) immediately post play, (PS3) 20-minutes post play, and (PS4) 40-minutes post play (see Figure 1).

### Mock MRI paradigm

The mock MRI is a non-social neutral stressor designed to evaluate reactivity to a situation with significant sensory components, including simulated MRI machine noises and relative confinement inside the scanning bore [55]. Participants were then scanned in a real MRI machine as part of a separate study protocol. A total of four salivary samples were taken, (MS1) approximately 15 minutes after arrival at the imaging facility just before exposure to the mock MRI, (MS2) 20 minutes after the start of the mock, (MS3) 40 minutes after the start of the mock, (MS4) taken just before the following real MRI scan (see Figure 1).

### TSST-C paradigm

The TSST-C is a well validated negative social stressor in which participants are asked to make a public presentation to neutral raters. The Trier Social Stress Test [56] was originally developed for adults and later adapted to children. In the child version, participants have 5 minutes of preparatory time and then finish a story prompt for 5 minutes in front of an audience composed of two adult raters. They are told that their presentation will be compared to those of all the other participants. After the 5-minute presentation, participants are asked to serially subtract a number determined by the child’s age and ability level. Four saliva samples were collected, including (TS1) 10 minutes after arrival, immediately before the TSST-C, (TS2) on completion of the TSST-C, (TS3) 20 minutes after completion of the TSST-C, and (TS4) 40 minutes after completion of the TSST-C (see Figure 1). Additional samples were collected as part of the TSST-C protocol [31], but were not part of the current investigation.

### STAIC and MASC administration

All three studies included a saliva sample taken after exposure to the stressor. The STAIC and MASC were administered to participants during the rest period immediately following this sample. Parents were present during administration of the STAIC and MASC, but were not permitted to assist their child. Research personnel carefully avoided referencing other terms used on the measures when answering participant questions. For example, the word nervous was avoided when describing what jittery means, as nervous appears later in the measure. The use of multiple measures with each participant allowed for better characterization of anxiety along multiple axes; the MASC focusing on anxiety subtypes, while the STAIC explored temporal characteristics.

### Cortisol sampling protocol

Established salivary collection protocols were carefully followed, including using consistent collection materials and methods, controlling the intake and time of drinks, food, and prescription medications, as well as using standardized procedures [54,66]. The basic procedure involved giving the participant Trident® Original Sugarless chewing gum to act as a salivary stimulant. The child then emitted saliva into a collection tube via passive drool until a total of 1 mL had been collected. Samples were stored in a -20°C freezer and prior to assay were thawed and centrifuged at 6,000 rpm for 10 minutes to separate the aqueous component from mucins and other suspended particles. Assays were performed using coated-tube radioimmunoassay kits (Siemens Medical Solutions Diagnostics, Los Angeles, CA, USA).

### Statistical analysis

Between-group analyses for the autism and typically developing groups were performed across demographic (age and IQ) and dependent variables of interest (STAIC and MASC anxiety) using independent two-sample t tests if the assumption of normality held true; otherwise, the equivalent non-parametric test was used. Pearson product correlations were performed to assess the association between self-reported anxiety and parent-reported social functioning (SRS) and physiological levels (cortisol values), respectively. Statistics were analyzed using SPSS version 21 (SPSS, Chicago, IL, USA).

## Results

**Figure 2:**
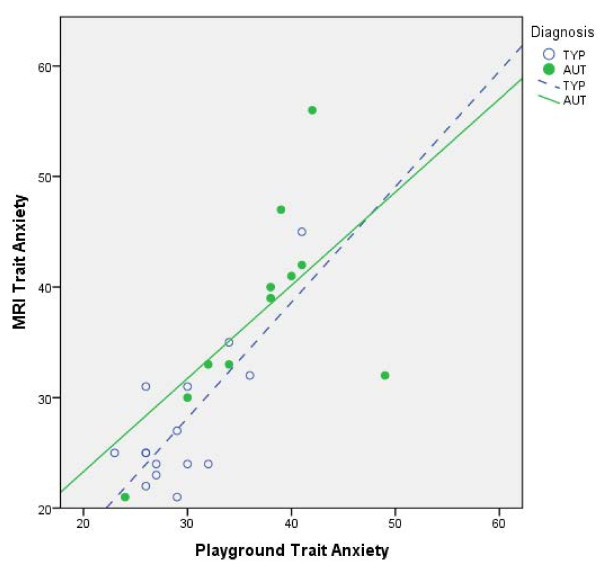
Association between playground and MRI trait anxiety in children with autism and children with typical development.

The primary aims of the study were to examine: (1) the longitudinal consistency of self-reported state and trait anxiety measures used with children with autism; (2) the relationship between both types of self-reported anxiety to parent-reported functioning; and (3) the association between reported anxiety and physiological stress responses. Self-reported anxiety was investigated across the two groups revealing significant differences between the typically developing and autism groups in terms of trait anxiety measured by the STAIC (see Table 2), with the autism group reporting significantly higher levels of trait anxiety in the playground and mock MRI conditions. In regards to the evaluation of long-term stability of self-reported anxiety, participants with autism demonstrated consistency in their reported anxiety between the playground and MRI conditions for both state (r = 0.685, *P* = 0.020) and trait (r = 0.607, *P* = 0.048) anxiety (see Figure 2). The playground and TSST-C anxiety reports, however, did not share this relationship (*P* >0.05). Typically developing children showed very strong consistency between the playground and MRI for both state (r = 0.931, *P* <0.001) and trait (r = 0.776, *P* = 0.001) anxiety. Trait anxiety between playground and the TSST-C was also highly correlated (r = 0.854, *P* = 0.031). Analyses between MRI and TSST-C reported anxiety were not made due to limited sample size of participants shared between the two conditions. Thus, the STAIC appears to be a stable measure for use in children with autism in some stress conditions (Figure 2).

**Table 2:** Anxiety measures (IQ controlled)

**Measure**	**Autism N**	**Autism mean**	**Autism SD**	**Typical N**	**Typical mean**	**Typical SD**	**F**	***P *** **value**	**Eta**^**2**^	**Power**
Playground state	19	30.79	6.99	21	26.52	5.13	0.55	0.46	0.114	0.579
Playground trait	19	38.95	8.55	21	30.29	4.74	15.43	<0.001	0.298	0.974
Mock MRI state	11	28.50	7.00	15	26.94	6.04	0.16	0.69	0.015	0.094
Mock MRI trait	11	37.50	8.92	15	28.31	6.72	6.55	0.017	0.272	0.850
TSST-C state	13	32.00	351	16	31.06	4.80	0.259	0.615	0.013	0.088
TSST-C trait	13	38.77	4.94	16	32.13	5.61	2.02	0.17	0.293	0.897
MASC total	19	59.61	13.76	21	47.86	8.59	6.80	0.01	0.223	0.895

*MASC* Multidimensional Anxiety Scale for Children, *TSST-C* Trier Social Stress Test - Child Version.

Self-rated anxiety on the MASC was also found to be significantly different between typically developing children and those with autism (See Table 2). Comparisons were also made between self-report state and trait anxiety using the STAIC and the MASC self-report anxiety scales. For the playground condition, overall state anxiety was moderately related to the MASC social anxiety subscale (r = 0.376, *P* = 0.026), while trait anxiety was strongly related to the same subscale (r = 0.716, *P* <0.001). There were also relationships between the coping subscale and both state (r = 0.335, *P* = 0.046) and trait (r = 0.396, *P* = 0.017) anxiety in the playground paradigm. Additionally, strong correlations were observed between the MASC social anxiety subscale and trait anxiety at both the MRI (r = 0.524, *P* = 0.006) and the TSST-C (r = 0.628, *P* = 0.012).

An additional relationship was found between anxiety in the playground condition and overall social functioning level measured by total score on the parent SRS measure for both state (r = 0.453, *P* = 0.004) as well as trait (r = 0.399, *P* = 0.012) anxiety. These results suggest that children with lower levels of overall social ability experience heightened anxiety in various situations when compared to their higher functioning peers. Similarly, the SRS Total Score was also correlated with MRI trait anxiety (r = 0.495, *P* = 0.009) and TSST-C trait anxiety (r = 0.549, *P* = 0.002). This demonstrates a relationship between parent-reported social functioning and child self-reported anxiety across conditions.

In regards to salivary cortisol levels, children with autism showed significantly higher activation in response to the playground social stressor (previously reported in [35]), and to the mock MRI (F(1, 27) 5.106, *P* = 0.03) when compared to typically developing children. In the TSST-C, however, children with autism showed a lack of responsivity compared to their typical peers, as previously reported [31]. Correlational analyses using Pearson product indicated a lack of association between cortisol levels at any given time point and self-reported anxiety (all *P* >0.05). Within group comparisons between the equivalent saliva samples and self-report anxiety showed that in children with autism there was no correlation between cortisol and anxiety on any of the stressors. Typically developing children, however, did show some relationship between cortisol and anxiety at certain time points. For example, salivary cortisol at arrival in children participating in the MRI condition correlated with trait anxiety (S1, r = 0.507, *P* = 0.045), whereas in the TSST-C condition arrival cortisol correlated with state anxiety (S1, r = 0.668, *P* = 0.006, and S2, r = 0.661, *P* = 0.007).

## Discussion

The current investigation explored the long-term stability of self-reported anxiety in autism as well as associations to parent-reported functioning and physiological stress responses. The findings reveal large differences between the typically developing and autism groups in trait anxiety and MASC subscales, providing further support that anxiety is a significant and common condition in children with autism (see, for example, [5-10]). The relative stability of self-report anxiety between the playground and mock MRI conditions indicates that children in this age group are potentially able to consistently report their persistent (trait) anxiety in stressful situations. Including basic self-report questionnaires, such as the aforementioned measures, under novel or changing situations may help to identify children who are routinely worried or anxious.

The lack of correspondence with the TSST-C reported anxiety may be due, in part, to differences in stress response, as the TSST-C failed to evoke a physiological response in children with autism [31,67]. As more fully described [30,31], children with ASD generally do not perceive the TSST-C to be stressful. In order to trigger the neuroendocrine cascade resulting in a release of cortisol, a situation must be perceived as stressful [25,26]. Children with ASD may interpret the social evaluative paradigm as being simply a cognitive exercise and thereby not respond in a typical manner. Alternatively, due to diminished social motivation [68], children with ASD may be less provoked by the evaluative nature of the task thereby contributing to a lower cortisol response and diminished endorsement of anxiety.

Moreover, the physiological stress under which a child with autism is requested to rate their general anxiety may influence their response. In other words, under conditions in which they exhibit physiological arousal as demonstrated by cortisol elevation they may be better able to rate their trait anxiety as being elevated, while under conditions in which they do not demonstrate physiological arousal such as the TSST-C, children with autism may appraise their overall anxiety as being lower. Interestingly, the children with autism did not consistently endorse state or current anxiety during the time of the stressor. It may be that children with autism are unable to discern current states of functioning yet are able to endorse enduring states of being. Sensory sensitivity frequently found in individuals with autism may be interfering with their ability to assess themselves cognitively during an experience. In other words, their sensory reactivity may be paramount and prevent them from being able to adequately identify internal states. Since this is merely conjecture, additional research is needed in this area. Our hypothesis that self-reported anxiety would be consistent across the different study paradigms was therefore only partially correct as consistency was only displayed for trait anxiety and only when the paradigm provoked a stress response.

External social cues related to autism symptoms, as well as direct cognitive awareness of one’s own impairments are both potential sources of anxiety for individuals with autism. Specific autism traits such as insistence on sameness [69] and restricted interests [70] have also previously been shown to relate directly to anxiety. The correspondence between parent-reported functioning and child anxiety levels reinforces the notion that a relationship exists between autism symptoms and anxiety [13]. Interventions targeting anxiety have demonstrated improvements in autism symptoms [71], indicating that this relationship may be reciprocal. It is thus important to consider children’s level of social function when evaluating for symptoms of anxiety in this population. In addition to core autism symptoms, anxiety also serves as a major source of challenge for parents [15], thereby warranting assessment and recommendations to assist families with coping in situations that contribute to excessive worry, avoidance, or discomfort. As cognitive ability and anxiety have a complex relationship, this finding is restricted to individuals with normal cognitive ability in addition to autism, and future investigations in populations with a broader range of functioning are needed. Our hypothesis that parent report of lower social ability would be associated with higher self-reported anxiety is thus supported, but only within this higher functioning cohort.

Our final hypothesis, that physiological stress measures would be associated with self-report anxiety, proved incorrect, as the data showed no such relationship. Anxiety and physiological stress, while often deeply intertwined, are distinct constructs that may be individually provoked under specific circumstances. The lack of correlation between salivary cortisol and self-report anxiety across all three stressors provides support for the distinctiveness of these constructs in children with autism [31]. In typically developing children, a positive correlation was present between reported anxiety and cortisol during arrival (prior to the stressor) for both the MRI and TSST-C conditions. Initial arrival for a novel activity can easily provoke both feelings of anxiety as well as physiological arousal. Additionally, the reduced consistency between cognitive appraisal and physiological response noted above may be a result of the HPA dysfunction commonly found in children with autism [72].

These findings demonstrate that children with average cognitive ability in addition to autism may be able to accurately assess their personal anxiety levels in contrast to some reports that question the usefulness of such measures [41,42], or show altered affective report [40]. These results build on evidence that adolescents with autism are able to self-report psychiatric symptoms, including anxiety [52]. While it remains to be seen in children with autism, under some conditions adolescents with the disorder may be more accurate reporters regarding their own mood dysregulation than their parents [52]. Taken together, these studies support the use of multi-informant measures to gain an enhanced understanding of perceived internal state (self-report), behavioral presentation (parent and teacher report) and symptom profile (clinical assessment) in individuals with autism. Additionally, informant discrepancies that arise in the process may offer valuable, clinically-relevant information in addition to that provided by self-report or parent report alone [50]. Additionally, the present study indicates that social functioning level is a prime indicator for the anxiety levels of children with autism.

Clinical implications of this study include providing evidence of long-term stability of the trait portion of the STAIC in this population across consistent conditions. Support is also present for using parent-reported functioning measures, such as the SRS, as an alternate method of assessing anxiety risk. As the STAIC and MASC are normalized for typically developing individuals, closer inspections through reliability and validity studies, which have been conducted in typically developing youth [73], are needed for individuals with autism.

## Conclusions

Due to the frequency of occurrence and deleterious effects on quality of life, addressing anxiety in children with autism is highly important. Interventions to reduce anxiety are often warranted and may be especially prudent in populations with lower social functioning, particularly those with self-assessed anxiety relating to social interaction or coping ability. Cognitive behavioral therapy (CBT) has been shown to be effective in children with autism to ameliorate anxiety symptoms [71,74,75], and autism specific anxiety interventions are under development [76]. Use of STAIC trait anxiety provides an easily administered indicator of whether CBT or other intervention methods for anxiety are appropriate for a given child. Self-report measures in adolescents with autism and anxiety have corroborated treatment gains observed by others following CBT [53] suggesting that children may similarly be able to identify changes over time in their perceived trait anxiety.

Additionally, the current results demonstrate the lack of an association between stress, as measured by salivary cortisol, and self-reported anxiety in children with autism. While this has previously been established in regards to the TSST-C, this study extends the finding to stressors that induce physiological reactivity in children with autism. This is an important consideration for research involving biological stress measurements as it strongly indicates that anxiety cannot be used as a predictor of biological reactivity to the environment in autism. Some evidence exists of a relationship in typically developing children between salivary cortisol at arrival and self-report anxiety, and future research would be well guided to examine this further.

Despite these interesting findings, there are some limitations to acknowledge. The sample size was somewhat moderate for a few of the experimental conditions. Furthermore, the age range was narrow due to the developmental play paradigm and exclusion criteria for puberty. While diagnostic and study inclusion criteria in the field have changed [1], the children in the sample were diagnosed, according to DSM-IV criteria, as having strictly defined autistic disorder [3]. Generalizability of the study is thus limited to male children with relatively unimpaired cognitive ability within a rather narrow age range. An additional factor limiting generalizability of this study was the lack of socioeconomic status data available for participants. Nevertheless, we view the rigorous clinical characterization of our participants as a strength of the study. Finally, because the nature of the study is largely exploratory, we acknowledge that the possibility of type 1 error is elevated due to the number of analyses conducted.

In summary, many children with autism exhibit persistent anxiety that can be captured via self-report measures. Furthermore, children’s anxiety is directly related to their level of social impairment. While no relationship was found between salivary cortisol reactivity and self-report anxiety, it appears that reported anxiety remains consistent between conditions that evoke similar physiological responses. Future research designed to identify children with enduring and impairing anxiety remains an important pursuit. Associations with social, perceptual, and cognitive functioning may identify subgroups at risk and in need of targeted treatment for anxiety in autism.

## Competing interests

The authors declare that they have no competing interests.

## Authors’ contributions

DMS collected and interpreted participant data for both self-report and parent-report forms, as well as physiological measures, and also wrote a major portion of the finished manuscript. BAC analyzed and interpreted participant data for both self-report and parent-report forms as well as the physiological data, and also made major contributions and edited the finished manuscript. Both authors read and approved the final manuscript.
